# Indents

**Published:** 1911-03-15

**Authors:** 


### INDENTS.

☞Brief Articles of Technical or Scientific Value will receive notice and comment. Contributions invited.

*The Ideal School Clinic.* Let us now look again at our public school problem. In the first place, I believe that this service is woman's work. Let us suppose that this picture exists, and then we will discuss its possibilities. In a city of one hundred thousand people, a room in one of the largest school buildings is equipped with fifteen dental chairs, dental brackets, compressed air, wash-bowls in center of room, sterilizers, etc. Beside these chairs stand fifteen women all dressed in white. Two of them are dental graduates and are licensed to practice dentistry. The other thirteen are assistants, and are being trained as dental nurses. The duties of the two women graduates are to relieve the sufferings from toothache of any of the poor children in the schools, and also to fill as many cavities with cement, alloy and gutta percha as they are able to do. The thirteen nurses are supplied with patients by teachers in the various rooms, who select those most worthy and least able to pay for dental services,—these children to be put through a system of cleanliness and education to prevent further dental troubles. Every two months each child appears for inspection; the teeth are thoroughly polished with the orange-wood sticks; special instructions in brushing given; brushes furnished to those unable to buy; a card system kept for reference; in fact, every effort made to establish among the children the habit of maintaining clean mouths. At least once a month one of the graduates, or some one appointed for the purpose, gives a talk or lecture on oral hygiene and the care of the teeth before the classes in the school rooms.

With such a system prevailing there would be, in five years' time, some chance of getting control of the flood of decay, and I believe in ten years' time the percentage figures could almost be reversed. But the Boards of Aldermen, the Boards of Apportionment, the Boards of Education, are like the man from Missouri—they must be shown. Demonstrate such a system of cleanliness and education even in a small way, for a period of two years, and there would be little trouble to secure appropriations to maintain the work, but the initial expense must be bridged by some philanthropic person or supported by the local dental society. This could be started in a small and modest way.

I believe it is possible to secure the services of the dental nurses free for the first year, as it would be a period of instruction and education for them, fitting them for good salaried positions in dental offices. Arrangements could be made so that they could attend certain lectures, either in hospitals with the nurses' course, in the larger cities at the dental colleges, and also by men appointed by the local dental society to talk to them on oral hygiene.

If this plan can be developed on practical lines, we will have the question of our public clinics for school children solved in a few years. We would have trained assistants available for the establishment of a chair for prophylaxis in every dental office. The children would be taking their lessons on oral hygiene home with them, and would in turn educate their parents on the importance of clean and healthy mouths, and instead of our dental colleges turning out but four thousand graduates a year, the country would need ten thousand to take care of the great ninety per cent. who need their children to tell them how to maintain their teeth and health by cleanliness.—Dr. Alfred C. Fones in *Items of Interest*.

*Prophylactic Treatment of Fissures and Pits in the Enamel.*

The prophylactic treatment of the teeth by mechanical or chemical means has little or no influence upon the enamel fissures and pits which are found especially in the bicuspids and molars. These deep fissures and pits which are observed even in children's teeth before or immediately after eruption, showing that they are normal and not due to any outside influences, are so deep and narrow that they cannot be reached by the toothbrush. Neither is the necessarily transient action of an antiseptic sufficient to combat the fermentation of food debris which produces such fatal results in these as it were natural culture tubes. The formation of lactic acid in these pits and fissures remains unchecked, and soon carious cavities result. In fact the majority of such cavities that are observed in daily practice have their origin in these pits and fissures, and at twenty years of age very few patients exhibit an intact condition of the fissures. Solbrig therefore advocates the filling of all fissures and pits with some plastic material immediately after the eruption of the teeth. This treatment involves various advantages: It is easily and quickly applied without causing pain or necessitating the sacrifice of hard structure, and saves the patient much subsequent expense. It is specially indicated in first molars, the preservation of which is of such cardinal importance. At the period of the eruption of the first molars the child is negligent of all oral hygiene, and very much afraid of pain at any time. By these preventive measures the teeth can be kept free from caries until the patient realizes the full importance of oral hygiene. In already carious cavities the method of procedure must needs be a different one; this also applies to the third molar, in which, owing to the peculiar anatomical conditions of this tooth, *i. e.*, the lodgment of food under the fibro-mucous gingival tissue, frequently causes carious fissures upon eruption.

The preservative treatment of enamel pits and fissures consists in the following procedure: With an explorer all food debris is most carefully removed from the fissures and pits, and the latter are washed with alcohol and dried with warm air. Very liquid cement is introduced, extreme care being taken to secure absolute closure. Despite its subsequent discoloration, preference is given to the oxy-phosphate of copper cement, owing to its indisputable antiseptic properties, although other cements will produce the same result, there being no wear on these extremely minute fillings. It is sometimes noted that these fillings are supplied by nature itself in the form of tartar deposits. This rational form of treatment of enamel pits and fissures will preserve the first molar better than any other prophylactic means, thereby safeguarding the equilibrium of articulation and the patients' general health.—M. O. Solbrig in *Cosmos.*

*To make a hard Plaster Model.* One part of Portland cement mixed with twenty parts of plaster of Paris will make a perfectly hard model cast.—*Chemist and Druggist.*

*Economy in Hypodermic Needles.* Hypodermic needles which have become occluded owing to the deposition of material derived from the fluids used for injection are often carelessly thrown away. All that is necessary to be done in order to restore the brightness of the external surface of such needles, as well as to cleanse them internally by dissolving out the precipitated material, is to boil them for a period of ten minutes in a solution of sodium bicarbonate in water. The economy of such a procedure is obvious in view of the cost of these needles.—*Medical Record* per *Dental Surgeon.*

*Germicide for Pulpless Tooth-roots.* As a germicide to prevent infection, or to destroy existing infection in pulpless tooth-roots, Dr. H. C. Register, of Philadelphia, (*Dental Cosmos,* September 1910), recommends the following:

℞—Iodoform,

Zinc oxid.................................aa ℥ss

Formaldehyd...............................m x

Sodium benzoate...........................gr. iv

Oil Cassia................................dram ivss

M

Trituate the iodoform and zinc oxid on an ointment slab intimately, then gradually add and thoroughly incorporate the oil of cassia. Dissolve the sodium benzoate in the formaldehyde and mix it thoroughly with the iodoform and zinc oxid. This forms a smooth ointment, light yellow in color with the odor of cinnamon, a pungent taste, and a slight solubility in water. A second preparation is made from this in a liquid form by adding to about two drams, two drams each of chloroform and alcohol. The fluid preparation penetrates into dehydrated dentin more readily during treatment. For closing the canal after completing treatment, a small pellet of the ointment is packed into the pulp canal, and a gutta-percha cone dipped into chloroform is pressed into the ointment in the canal, and thoroughly packed, or a small amount of the ointment may be incorporated in a mix of zinc phosphate cement with which the canal is filled.

*Gum Lotions for Odontalgia.*

I.

℞—Tincture of iodin,
Tincture of aconite, āā dram ijss. M.

II.

| | |
|---|---|
| ℞—Tincture of iodin, | dram j |
| Tincture of aconite, | dram j |
| Crystalline synthetic guaiacol, | dram jss. M. |

III.

| | |
|---|---|
| ℞—Tincture of iodin, | dram jss |
| Tincture of aconite, | dram jss. |
| Chloroform, | dram j. M. |

*Pulp Applications for Odontalgia.*

I

| | |
|---|---|
| ℞—Tincture of benzoin, | dram ijss |
| Oil of cloves, | m xlv. M. |

II.

℞—Solution of formaldehyd, U. S. P. — dram ijss
Oil of geranium, — m xxx. M.

III.

℞—Chloroform, — dram ijss
Creasote, — dram ijss
Tincture of opium, — dram ijss
Tincture of benzoin, — ℥j. M.

IV.

℞—Chloral hydrate, — gr. lxxv
Cocain hydrochlorid, — gr. xv
Pulverized camphor, — gr. lxxv
Alcohol, — gtt. x. M.

V

℞—Tincture of benzoin, — dram jss
Chloroform, — dram j
Carbolic acid crystals, — gr. xxx. M.

Or the cavity may be filled with a paste of the following composition:

℞—Cocain hydrochlorid, — gr. 1-7
Morphin hydrochlorid, — gr. 1–7
Oil of cloves, q. s. to make a paste, — M.

—*New York Med. Journal.*

*Mouth-washes for Inflamed Gums.* The two following mouth-washes are recommended for inflamed gums. All tartar and foreign matter is first removed from the teeth, for unless the cause is removed, very little improvement will occur.

Potass. chlorat., — dram ij
Glycer. boracis, — dram v
Aquae q. s. ad — ℥x

*Sig.*—To be used frequently.

As a soothing and antiseptic mouth-wash:

Acid. salicylic, — dram jss

Spirit, rect., — dram ij

Aq. camphorae, q. s. ad. — ℥viij

—*Chemist and Druggist.*

*Application of Adrenalin to the Gums.* Adrenalin, when applied to the gums on cotton, whether to stop bleeding or, as has been recommended in the preparation of cavities, to control the saliva exuding from the mucous glands at the necks of the teeth, should be used with great caution. The cotton with the adrenalin solution should be thoroughly squeezed to remove any excess before applying. If adrenalin, used to stop bleeding, is accidentally swallowed, alarming symptoms may develop, not unlike epileptic seizures, and the patient may remain in a collapsed condition for some hours after. Specially prepared adrenalin gauze is now made, and this should be used in tamponing the alveolar socket of a tooth which has been extracted, and in which the bleeding is persistent. —*Chemist and Druggist.*

*Report of Reading, Penna., Public School Dental Inspection, June, 1910.*

| Age | No. Pupils | Greenstain | Gums Abnormal | Tartar | Occlusion Abnor. | Mouth Breathing | Atrophy of Teeth | Permanent Teeth Cavities | Temporary Teeth | Putrescent Pulps | Exposed Pulps | Extracted Permanent Teeth | Use of Brush | Previous Dental Work |
|---|---|---|---|---|---|---|---|---|---|---|---|---|---|---|
| 5 yrs. | 28 | 21 | 1 | 0 | 0 | 0 | 0 | 40 | 127 | 3 | 4 | 0 | 12 | 3 |
| 6 " | 989 | 521 | 43 | 57 | 39 | 25 | 17 | 1309 | 3662 | 249 | 177 | 5 | 305 | 63 |
| 7 " | 1107 | 625 | 51 | 90 | 110 | 20 | 42 | 2240 | 4260 | 300 | 164 | 34 | 395 | 69 |
| 8 " | 1067 | 664 | 79 | 150 | 175 | 21 | 39 | 2936 | 2981 | 239 | 186 | 29 | 535 | 91 |
| 9 " | 1134 | 733 | 117 | 215 | 191 | 29 | 26 | 3276 | 2136 | 236 | 213 | 81 | 617 | 157 |
| 10 " | 1044 | 776 | 126 | 216 | 203 | 30 | 27 | 3262 | 925 | 195 | 216 | 151 | 637 | 157 |
| 11 " | 1058 | 790 | 132 | 265 | 196 | 36 | 33 | 3630 | 434 | 184 | 207 | 187 | 698 | 191 |
| 12 " | 1100 | 802 | 172 | 331 | 262 | 24 | 60 | 4486 | 108 | 230 | 253 | 264 | 754 | 263 |
| 13 " | 910 | 665 | 150 | 336 | 233 | 22 | 41 | 4879 | 35 | 183 | 193 | 230 | 561 | 222 |
| 14 " | 325 | 242 | 41 | 141 | 108 | 18 | 12 | 1816 | 26 | 49 | 71 | 79 | 241 | 111 |
| 15 " | 122 | 55 | 14 | 49 | 27 | 8 | 8 | 568 | 10 | 22 | 25 | 29 | 76 | 35 |
| 16 " | 39 | 15 | 3 | 14 | 9 | 3 | 3 | 102 | 2 | 4 | 7 | 5 | 16 | 5 |
| 17 " | 2 | 1 | | 2 | 1 | | | 4 | 1 | | 1 | | 2 | 2 |
| Totals | 8925 | 5910 | 925 | 1866 | 1554 | 236 | 308 | 28,548 | 14,707 | 1894 | 1717 | 1094 | 4849 | 1369 |
| | | 66 1/5% | 10 1/3% | 21% | 17 2/5% | 1/4% | 1/3% | Average 3 1/5% | Average 1 3/5% | 21 1/5% | 19 1/3% | 12 1/5% | 54 1/3% | 15 1/3% 84 2/3% |
| | | | | | | | | | | Less 5 | th ove | r 5 h o | ver 8th | |

—*Dental Brief*

